# Morphometric Study of *Mus musculus*, *Rattus norvegicus*, and *Rattus rattus* in Qatar

**DOI:** 10.3390/ani11082162

**Published:** 2021-07-22

**Authors:** Md Mazharul Islam, Elmoubashar Farag, Ahmad Mahmoudi, Mohammad Mahmudul Hassan, Muzzamil Atta, Ehsan Mostafavi, Ismail Alnour Alnager, Hassan Ali Farrag, Gaafar El Awad Eljack, Devendra Bansal, Mohamed Haroun, Randa Abdeen, Hamad Al-Romaihi, Abdul Aziz Al-Zeyara, Sowaid Ali Almalki, Zilungile Mkhize-Kwitshana

**Affiliations:** 1 Department of Animal Resources, Ministry of Municipality and Environment, Doha P.O. Box 35081, Qatar; maabdalla@mme.gov.qa (M.A.); rahassan@mme.gov.qa (R.A.); amzeyara@mme.gov.qa (A.A.A.-Z.); sowaidalmaliki@hotmail.com (S.A.A.); 2 School of Laboratory Medicine and Medical Sciences, College of Health Sciences, University of KwaZulu Natal, Durban 4000, South Africa; 3 Ministry of Public Health, Doha P.O. Box 42, Qatar; dbansal@moph.gov.qa (D.B.); mismail_99@yahoo.com (M.H.); halromaihi@moph.gov.qa (H.A.-R.); 4 Department of Biology, Faculty of Science, Urmia University, Urmia 5756151816, Iran; a.mahmoudi@urmia.ac.ir; 5 Faculty of Veterinary Medicine, Chattogram Veterinary and Animal Sciences University, Khulshi, Chattogram 4225, Bangladesh; miladhasan@yahoo.com; 6 College of Animal Production, University of Bahri, Khartoum 11111, Sudan; 7 Department of Epidemiology and Biostatistics, Research Centre for Emerging and Reemerging Infectious Diseases, Pasteur Institute of Iran, Tehran 1316943551, Iran; mostafaviehsan@gmail.com; 8 National Reference Laboratory for Plague, Tularemia and Q Fever, Research Centre for Emerging and Reemerging Infectious Diseases, Pasteur Institute of Iran, Akanlu, Kabudar Ahang, Hamadan 6556153145, Iran; 9 Rayan Municipality, Ministry of Municipality and Environment, Doha, Qatar; ismail1956a@gmail.com; 10 Qatar Pest Control Company, Doha P.O. Box 6319, Qatar; hfarrag52@yahoo.com (H.A.F.); gaafarawad@yahoo.com (G.E.A.E.); 11 School of Life Sciences, College of Agriculture, Engineering & Science, University of KwaZulu Natal, Durban 4000, South Africa; mkhizekwitshanaz@ukzn.ac.za; 12 South African Medical Research Council, Cape Town 7505, South Africa

**Keywords:** rodents, small mammals, commensal species, morphometry, Qatar

## Abstract

**Simple Summary:**

Rodents are the most abundant and diversified group of mammals. These animals show genetic and physical diversity in different ecosystems of the world, including the desert ecosystem. The current study was undertaken to check the morphometric pattern of three commensal rodent species, viz, *Mus musculus*, *Rattus norvegicus*, and *Rattus rattus*, in Qatar. One hundred forty-eight rodents were captured and studied for body and cranio-mandibular measurements. The study found *R. norvregicus* as the most prevalent rodent in Qatar. Most of the rodents were collected from Al Rayan municipality, were adults, and were from livestock farms. The rodents’ average body weights were 18.8 ± 2.2 gm, 264.3 ± 87.5 gm, and 130 ± 71.3 gm for *M. musculus*, *R. norvegicus*, and *R. rattus*, respectively. The average morphometric measurements of the external body and skull were normally distributed and can be used as a reference of *R. norvegicus* and *R. rattus* for Qatar.

**Abstract:**

The current study was undertaken to estimate the morphometric pattern of three commensal rodents, i.e., *Mus musculus*, *Rattus norvegicus*, and *Rattus rattus* in Qatar. One hundred forty-eight rodents were captured from different facilities throughout Qatar. The captured rodents were used to identify the external body and cranio-mandibular morphometry. The study found that *R. norvregicus* was the most prevalent (*n* = 120, 81%, 95% CI: 73.83–87.05). Most of the rodents were collected from Al Rayan municipality (*n* = 92, 62%), were adults (*n* = 138, 93.2%, 95% CI: 87.92–96.71), and were from livestock farms (*n* = 79, 49%, 95% CI: 41.02–57.65). The rodents’ average body weights were 18.8 ± 2.2 gm, 264.3 ± 87.5 gm, and 130 ± 71.3 gm for *M. musculus*, *R. norvegicus*, and *R. rattus*, respectively. The research found that the studied rodents are smaller than those of other countries such as Turkey, Tunisia, and Iran. The study of morphometry is a useful tool for the traditional identification of small mammal species, including rodents. The average morphometric measurements of the external body and skull were normally distributed and can be used as a reference of *R. norvegicus* and *R. rattus* for Qatar. A further comprehensive study is required to investigate the rodent population index, eco-friendly control program, and public health importance in Qatar.

## 1. Introduction

Rodents are the largest group of mammals, distributed on every continent of the world except Antarctica [1]. Globally, there are 2552 rodent species available, of which three species, i.e., house mice (*Mus musculus*), brown rat (*Rattus novegicus*), and black rat (*Rattus rattus*), occupy different habitats with higher density than other species of rodents [2,3]. These human commensals live in diverse ecosystems throughout the world, showing high morphological and genetic variation. For instance, the brown rat showed at least 13 evolutionary clusters globally [4]. Several evolutionary factors, such as climate and geography, predators, urbanization, and agricultural settlement, are behind these evolutionary changes [5,6,7]. The desert environment is also a factor for the phenotypic and genotypic evolutionary change of mammals. For example, fur coloration and its covariation with habitat have been reported for desert gerbils [8]. Genetic analysis and phenotypic and morphometric assessments provide unique ways of identifying different mammalian species and evaluating animal diversity evaluation [7,9]. The external and cranio-mandibular morphologies are valuable tools in the classification of rodent species. The bones of a skull have some variation between and within a mammalian species that lead their species or subspecies to a distinguished morphological identity [9].

The state of Qatar is a small country in the Arabian Peninsula, whose terrain comprises sand dunes and salt flats across a low barren plain [10,11]. The country has a dry, subtropical climate, with very low annual rainfall (33.1 mm in 2010 and 114.1 mm in 2015), intensely hot (42.7–48.1 °C) and humid (32–72% relative humidity) summer, and warm (10.7 °C) winter. Due to the climate and geography, agricultural practices are limited in Qatar [10,12]. Rodents have importance for animal and public health in this country [13]. Rodent-borne pathogens, such as *Coxiella* and *Toxoplasma*, are common causes of livestock abortion in Qatar [14]. *Taenia taeniaeformis*, *Toxoplasma godii*, and *Toxascaris leonina* were reported among pet animals [15,16]. Zoonoses that can be associated with rodents, such as *Escherichia coli*, *Giardia duodenali*, and *Hymenolepis nana*, were reported among human populations in this country [17,18]. Moreover, the zoonotic cestode, *Hymenolepis diminuta*, was identified among *R. norvegicus* in Doha city of Qatar [19,20]. The country has governmental [21] and non-governmental rodent control programs. Minimal research, however, has been done on rodents in this country [13,19,20]. There is no documented report of rodent identification guidelines, such as morphometry of rodents in Qatar. Therefore, the present research aimed to study three commensal rodents, such as *Mus musculus*, *Rattus norvegicus*, and *Rattus rattus*, to identify the specific species of the rodents and to understand their physical and behavioral characteristics that are potentially found in the Qatar.

## 2. Materials and Methods

### 2.1. Study Season, Area, and Rodent Collection

**Figure 1:**
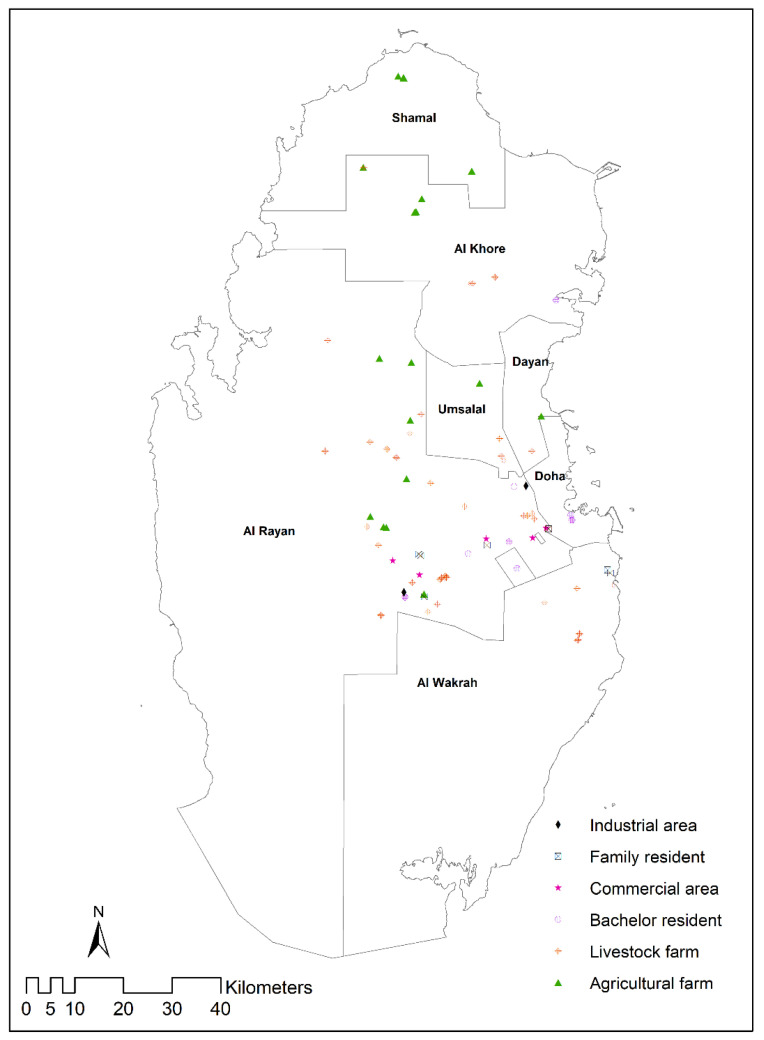
The map shows successful rodent trapping locations in different settings of Qatar.

A cross-sectional study was done from November 2019 to February 2020 as a part of routine pest control program in Qatar. A total of 250 traps were used, which include 150 single rodent traps (SRT) and 100 multi rodent traps (MRT). We used different types of baits such as bread (Arabian khubj), biscuits, potato chip, and cheese for capturing the rodents [22]. An SRT or MRT was used randomly, without targeting any specific rodent species or the species behavior. A water bottle containing 5% glucose was affixed to each trap to reduce dehydration and stress of the captured animals in the harsh Qatari environment. The trappings covered six facilities: family residents, bachelor residents, agricultural farms, livestock farms, industrial areas, and commercial areas throughout Qatar (Figure 1). The traps were set for a single night. Successful traps were collected in the morning and transferred at the earliest convenience to the veterinary laboratory, Doha, Qatar. A comfortable temperature was maintained (20–25 °C) in the transportation car and veterinary laboratory rodent room. The traps were washed with soap and pressurized water and air-dried to avoid any residual contamination and transmission from the previous rodent to the next.

### 2.2. Rodent Identification and Morphometric Assessment

The captured rodents were euthanized using 5% isoflurane inhalation for five minutes in a desiccator. After weighing with an electronic balance (Serial No. 057700082, Kern EG420-3NM, Kern & Sohn GmBH, Balingen, Germany), morphological appearance and external measurements were recorded as per species, age, sex, and pregnancy [22,23,24,25]. Rodent species were identified based on morphologic characteristics and measurements. The animals were assessed for sex (female or male) using external and internal aspects of reproductive organs such as testicles, penis, seminal vesicles, vagina, mammary teats, and possible pregnancy signs. For age detection, we only identified the adult rodents. Developed genital organs and pregnancy were the sign of an adult rodent. Additionally, we considered prominent temporal ridges and postorbital processes of the skull to determine a rodent as mature. The presence of a gravid uterus served as the indicator of pregnancy.

**Figure 2:**
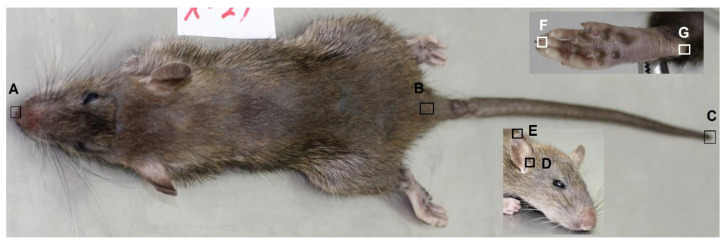
External view of a rodent body with linear measurement marks. General length (**A** to **C**, **C** is the last caudal vertebra), Tail length (**B** to **C**, **B** marks anus), Body (Head and body) length (**A** to **B**), Right ear length (**D** to **E**), and Right hind leg length (**F** to **G**).

**Figure 3:**
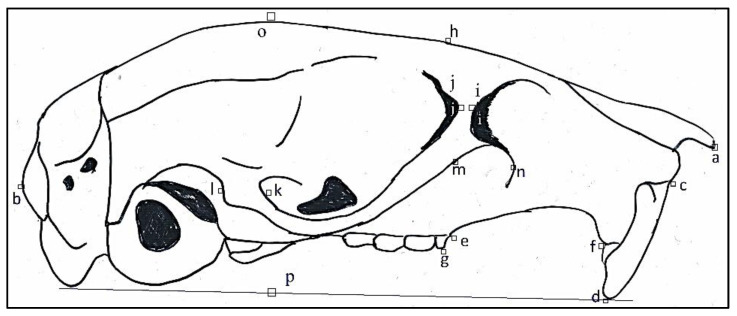
Lateral view of a rodent skull with linear measurements and identification marks. General cranial/Occipitonasal length (**a** to **b**), Length of upper incisor (**c** to **d**), Distance between upper incisor to alveolus molar tooth 1 (**d** to **e**), Length of diastema (**e** to **f**), Rostrum height (**g** to **h**), Breath of inferior ramus of zygomatic process of maxillary (**i** to **j**), Breath of base zygomatic process of squamosal (**k** to **l**), Breath of zygomatic plate (**m** to **n**), and General cranial height (**o** to **p**).

**Figure 4:**
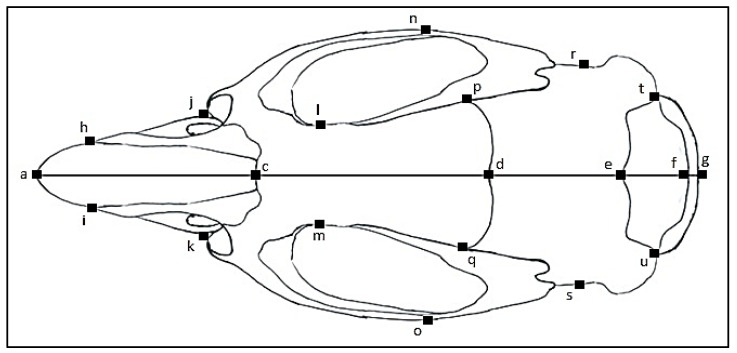
Dorsal view of a rodent skull with linear measurements and identification marks. Breath of nasal bones (**h** to **i**), Greatest rostrum breath (**j** to **k**), Smallest intraorbital breath (**l** to **m**), Zygomatic breath (**n** to **o**), Frontal bone width (**p** to **q**), Breath of brain cage (**r** to **s**), Interparietal bone width (**t** to **u**), Occipital bone length (**f** to **g**), Interparietal bone length (**e** to **f**), Parietal bone length (**q** to **u**), Frontal bone length (**c** to **d**), Nasal bone length (**a** to **c**).

**Figure 5:**
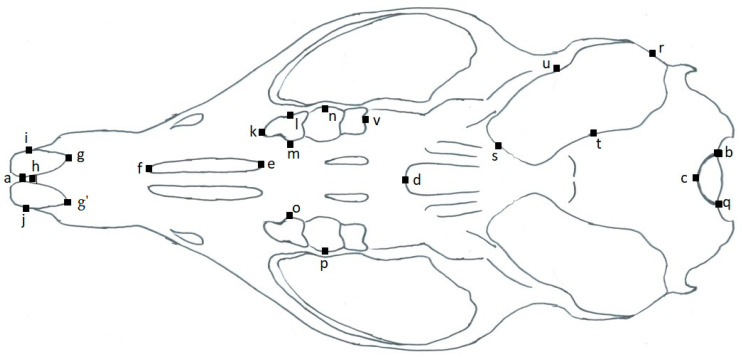
Ventral view of a rodent skull with linear measurements and identification marks. Condylobasala length (**a** to **b**), Henselion-basion distance (**h** to **c**), Henselion-palatial distance (**h** to **d**), Palatal foramen length (**e** to **f**), Smallest palatal breath (**m** to **o**), Upper cheek to teeth alveoli (**k** to **v**), Breath of upper dental arch (**n** to **p**), Breadth of molar tooth 1 (**m** to **l**), Width of upper incisor basal part (**i** to **j**), Width of the upper incisor apex part (**g** to **g’**), Tympanic bulla length (**r** to **s**), Tympanic bulla width (**t** to **u**), Foramen magnum width (**b** to **q**).

**Figure 6:**
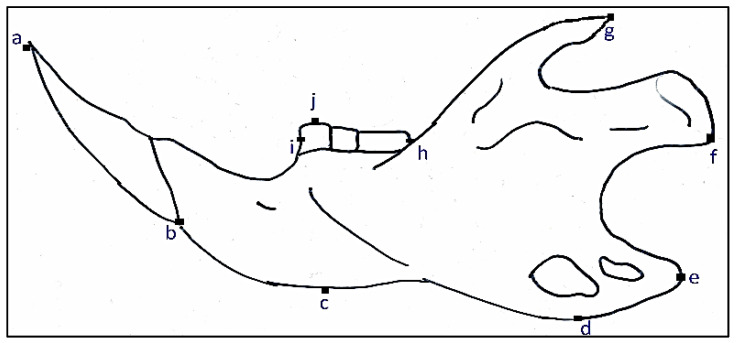
Lateral view of rodent mandible with linear measurements and identification marks. Length of lower incisor (**a** to **b**), Distance between lower incisor to coronoid process (**a** to **g**), Distance between lower incisor to condyloid process (**a** to **f**), Distance between lower incisor to angular process (**a** to **e**), Greatest jaw height (GJH) (**d** to **g**), Ramus to Molar tooth 1 (**c** to **j**), Distance between lower molar tooth 1 to molar tooth 3 (**h** to **i**), and Distance between lower incisor to molar tooth 1 (**a** to **i**).

Five standard external measurements were made for the animals using a ruler (Figure 2). Following the morphological characterization, the rodents were dissected, skulls were collected, cleaned, and dried according to the standard procedure [26]. The cranium and mandible morphometric variables were recorded using a digital caliper (TESA TWIN-CAL IP67, Hexagon, Switzerland) described previously [9,27,28,29] and illustrated in Figure 3, Figure 4, Figure 5 and Figure 6.

### 2.3. Statistical Analysis

The data were analyzed using statistical software StatSoft (2011) to study the descriptive analysis of the number of captured rodents and their morphometric variables that included mean, percentage (%), 95% confidence interval (CI), standard deviation (SD), skewness, standard error of skewness, kurtosis, and standard error of kurtosis. The data were tested with the Kolmogorov–Smirnov test, skewness, and kurtosis to validate the normality. If the skewness and kurtosis were outside −2 and +2, the measurement was considered significantly skewed or kurt [30,31]. The student *t*-test was performed to examine the variability of the morphometric traits among sex (female vs. male) and pregnancy (pregnant vs. non-pregnant). The chi-square (χ^2^) test was performed to examine the level of significance (*p* < 0.05) among the area (municipality) and trapping location types.

## 3. Results

### 3.1. Demographic Information

The study captured 148 rodents from all seven municipalities of Qatar (Table 1, Figure 1). A total of 79 rodents were captured by SRT and 69 rodents by MRT. The thirty-two MRT captured more than one rodent (2–5) at a time. Based on the morphologic and morphometric characters of the body and skull, three species of rodents were identified, i.e., *M. musculus*, *R. rattus*, and *R. norvegicus*. *R. norvegicus* comprised 81.1% (*n* = 120) of the total captured rodents, whereas *R. rattus* (*n* = 24) and *M. musculus* (*n* = 4) showed low density. Most of the collected rodents (*n* = 138, 93.2%) were adults. A major portion of the captured rodents was collected from Al Rayan municipality (*n* = 92, 62%). This municipality harbors all the three commensal species (*M. musculus* and *R. rattus*, and *R. norvegicus*), showing (χ^2^ = 21.02, *p* < 0.05) the highest density for *R. norvegicus* (*n* = 64). The majority of the rodents (*n* = 79, 49%) (χ^2^ = 35.29, *p* < 0.05) were collected from the livestock farms.

### 3.2. Morphometric Assessments of Rodents

The overall means of body weight, external morphometry, and cranio-mandibular variables per species are presented in Table 2, Table 3 and Table 4. Out of the 148 rodents, 108 rodents were dissected, comprised of 86 *R. norvegicus*, 18 *R. rattus*, and 4 *M. musculus*. The average body weight was variable among three rodent species (18.8 ± 2.2 gm, 264.3 ± 87.5 gm, and 130 ± 71.3 gm for *M. musculus*, *R. norvegicus*, and *R. rattus*, respectively). The skewness and kurtosis statistics of all the studied external body measurements of *R. norvegicus* and *R. rattus* were within −2 and +2. This indicated that the observed values were normally distributed. In general, the tail is longer than the length of the body and head of *M. musculus* and *R. rattus*, which is the opposite in *R. norvegicus*. Compared to the general length of a rodent, the ears and legs of *R. rattus* are longer than that of *R. norvegicus.* As the captured number of *M. musculus* was small, no further statistical comparative analysis could be considered on their body or cranio-mandibular measurements.

The *t*-test showed that there is no sexual or pregnancy-related dimorphism (*p* > 0.05) in any of the presented characteristics in the case of *R. norvegicus* (Table 5, Table 6, Table 7, Table 8, Table 9 and Table 10). However, the right ear length measurements showed that females have longer ears than males in *R. rattus*. Moreover, the mandibular characters, such as the length of lower incisors and the distance between lower incisor to coronoid process, lower incisor to condyloid process, lower incisor to angular process, ramus to molar tooth 1, and lower incisor to molar tooth 1 of *R. rattus*, were significantly higher in females than males (*p* < 0.05). In addition, the value of lower molar tooth 1 to molar tooth 3 was higher in the case of males than females in *R. rattus* (Table 7). Furthermore, the right hind leg was longer (*p* > 0.05) in non-pregnant than pregnant *R. rattus* (Table 8).

## 4. Discussion

The study of rodent demography is essential from ecological and public health perspective [32]. The present study identified three commensal rodent species in Qatar captured during routine pest control activities. These rodents have a cosmopolitan distribution and are mainly facilitated by anthropic activities [2]. Four species of rodents were reported previously in Qatar, viz., Arabian Jerboa (*Jaculus loftusi*, previously included in *Jaculus jaculus*), house mouse (*M. musculus*), brown rat (*R. norvegicus*), and black rat (*R. rattus*) [13,19,20,33]. *Jaculus loftusi* is a wild dipodid rodent that lives in the desert ecosystem, like the sandy and rocky places [34], so this species is not in the scope of the present study. However, the current study found that a significant component of commensal rodents in Qatar is *R. norvegicus*. This is supported by the previous reports [19,20], which captured only *R. norvegicus* during their studies in Qatar.

Our study revealed that most of the rodents were from livestock farms. The livestock farms are mostly made up of mixed livestock species with poor management and biosecurity [35], making an ideal place for rodents to colonize and why we captured a major part of rodents from these places. A previous study reported that over 75% of the livestock farms were infested with rodents, mainly by *R. norvegicus*, and the incidence of house mouse *M. musculus* was detected less in Qatar [13], which is congruent with the present study. Out of the 148 captured rodents, only four were *M. musculus*.

Traditional morphometry is a valuable tool for species identification in small mammals, including rodents [28,36]. The present study found the body weight and general body length of *R. norvegicus* as 264.3 gm and 398.5 mm, respectively, which were 259 ± 85.2 gm and 405 ± 54.7 mm, respectively, for the same species in Turkey [37]. In the case of cranial morphometry, the condylobasal length and the zygomatic breadth of *R. norvegicus* in the current study were 45.2 mm and 22.4 mm, which were 45.52 mm and 23.75 mm in the case of Turkey [37] and 46.84 mm and 21.64 mm in the case of Iran [38], respectively, for the same species and measurements. The overall body length of *R. rattus* in Turkey was 378.43, which was 324.4 mm for the same species of Qatar. The cranial length and zygomatic width of *R. rattus* in the current study were 37.2 mm and 18.2 mm, which were 39.15 mm and 19.86 mm, respectively, for Turkey [37] and 39.08 mm and 19.97 mm, respectively, for Tunisia, respectively for the same species and measurements [39].

Similarly, the body length of *M. musculus* in Qatar was 78.5 mm, which was 85.41 mm [24] and 88.0 mm [40] for the same species from different parts of Iran. Due to the small sample size (*n* = 4), we do not have strong support in the results of *M. musculus* morphometry. However, the overall body and cranial size indicate that the three studied rodent species in Qatar are comparatively smaller than the same species from the countries like Turkey, Tunisia, and Iran. This variation may be due to Qatar harsh environmental effects [6,7,8], which is supported by Bergmann’s rule [41]. Rodents of the colder environment are bigger in body size than the wormer environment [42,43]. This further highlights the necessity of performing traditional morphometry on the geographic population of rodents, specifically cosmopolitan species.

Based on the average general body and skull morphometric measurements, males were slightly larger than females, although there is no significant sexual dimorphism. This finding is supported by a previous study by Ventura and Lopez-Fuster [7]. However, the present study showed that the body and cranio-mandibular linear measurements of commensal rodents in Qatar were normally distributed for the two species, *R. norvegicus*, and *R. rattus.* Bodyweight and body and skull linear measurements distribution shape were approximately symmetric since the statistic of skewness measures were between −0.2 and 0.2 [30,31]. Normality analysis of the biometric traits can be considered typical characteristics of the two rodent species, *R. norvegicus* and *R. rattus*, in this country. To the best of the authors’ knowledge, such work is the first time in Qatar. Therefore, the current study can be used as a reference for morphometric measurements of the commensal rodents in this country, especially for *R. norvegicus* and *R. rattus.*

## 5. Conclusions

The current study estimated, identified, and characterized the morphometric variables of three commensal rodents in Qatar. The research identified that the commensal rodents of Qatar are comparatively smaller than the same species of some other countries, such as Iran, Tunisia, and Turkey. The is the first study on rodent morphometry in Qatar and even in the Arabian Peninsula. Due to geo-ecological similarities, the present study can be a reference study to rodent or small mammal identification in Qatar and other countries of the Arabian Peninsula.

## Figures and Tables

**Table 1:** Demographic characteristics of the trapped rodents.

Characters	*n* (% of Total Capture, 95% CI)
Trapping location (*n* = 148)	
Agriculture farm	31 (20.9, 14.69–28.39)
Bachelor residence	18 (12.2, 7.36–18.53)
Commercial area	11 (7.4, 3.76–12.91)
Family residence	11 (7.4, 3.76–12.91)
Industrial area	4 (2.7, 0.74–6.78)
Livestock farms	73 (49.3, 41.02–57.65)
Municipalities (*n* = 148)	
Al Khore	17 (11.5, 6.84–17.75)
Daayan	1 (0.7, 0.002–0.37)
Doha	10 (6.8, 3.29–12.07)
Rayyan	92 (62.2, 58.83–69.70)
Shamal	7 (4.7, 1.92–9.50)
Um Salal	8 (5.4, 2.36–10.37)
Wakrah	13 (8.8, 41.02–57.65)
Species (*n* = 148)	
*Mus musculus*	4 (2.7, 0.74–6.78)
*Rattus norvegicus*	120 (81.1, 73.83–87.05)
*Rattus rattus*	24 (16.2, 10.68–23.16)
Sex (*n* = 148)	
Female	75 (50.7, 42.34–58.98)
Male	73 (49.3, 41.02–57.65)
Pregnancy (*n* = 75)	
Pregnant	20 (26.7, 17.11–38.14)
Non-pregnant	55 (73.3, 61.86–82.89)
Age (*n* = 148)	
Adult	138 (93.2, 87.92–96.71)
Young	10 (6.8, 3.29–12.07)

**Table 2:** The external body linear measurements (mean ± SD) of the commensal rodents of Qatar.

Sl. No.	Parameters *	*Mus musculus* (*n* = 4)	*Rattus norvegicus* (*n* = 120)	*Rattus rattus* (*n* = 24)
1	Body weight	18.8 ± 2.2	264.3 ± 87.5	130.0 ± 71.3
2	General length	163.8 ± 4.8	398.5 ± 45.1	324.4 ± 80.0
3	Tail length	85.3 ± 4.1	191.4 ± 22.9	181.3 ± 39.0
4	Body length	78.5 ± 2.4	207.1 ± 23.0	143.1 ± 44.4
5	Right ear length	13.3 ± 1.7	18.9 ± 1.7	18.6 ± 2.1
6	Right hind leg length	16.5 ± 1.3	39.2 ± 3.6	32.4 ± 3.9

* The body weight was measured in grams and the rest of the parameters were measured in millimeters; *n*: Total observation, and SD: Standard deviation of mean.

**Table 3:** Cranial morphometric linear measurements (mean ± SD) of the commensal rodents of Qatar.

Sl. No.	Parameters *	*Mus musculus* (*n* = 4)	*Rattus norvegicus* (*n* = 86)	*Rattus rattus* (*n* = 18)
1	General cranial length	21.9 ± 0.4	46.8 ± 4.1	37.2 ± 2.7
2	Condylobasal length	21.3 ± 0.1	45.2 ± 4.1	35.5 ± 2.9
3	Henselion-basion length	18.7 ± 0.4	39.1 ± 3.6	29.4 ± 2.9
4	Henselion-palpation length	11.2 ± 1.3	22.4 ± 2.3	16.9 ± 1.7
5	Length of upper incisor	3.3 ± 0.6	7.5 ± 1.6	5.6 ± 1.0
6	Width of upper incisors, basal	2.1 ± 0.2	4.8 ± 0.6	3.5 ± 0.5
7	Width of upper incisors, apex	1.3 ± 0.1	3.3 ± 0.5	2.2 ± 0.4
8	Upper incisor to alveolus molar tooth 1	6.3 ± 0.4	14.4 ± 1.9	10.1 ± 1.4
9	Length of diastema	5.9 ± 0.4	13.4 ± 1.5	9.6 ± 1.2
10	Nasal bone length	7.5 ± 0.6	17.2 ± 1.9	12.6 ± 1.4
11	Breath of nasal bones	2.2 ± 0.4	5.2 ± 0.6	3.9 ± 0.3
12	Frontal bone length	7.1 ± 0.4	14.7 ± 1.3	12.2 ± 1.4
13	Frontal bone width	5.7 ± 1.1	10.9 ± 0.6	10.3 ± 1.1
14	Parietal bone length	7.3 ± 0.5	13.0 ± 1.1	11.2 ± 1.0
15	Breath of brain cage	9.8 ± 0.4	16.4 ± 2.1	16.2 ± 0.6
16	Interparietal bone length	3.2 ± 0.2	6.5 ± 0.7	5.5 ± 0.6
17	Interparietal bone width	6.7 ± 1.5	11.5 ± 1.0	10.7 ± 0.8
18	Occipital bone length	4.5 ± 0.4	6.0 ± 0.8	4.5 ± 0.4
19	General cranial height	7.4 ± 0.1	16.6 ± 1.5	13.7 ± 0.8
20	Rostrum height	6.3 ± 0.3	13.8 ± 1.3	10.8 ± 0.9
21	Rostrum breathe	3.5 ± 0.1	9.0 ± 1.0	6.5 ± 0.7
22	Smallest interorbital breadth	3.4 ± 0.3	6.8 ± 0.5	5.8 ± 0.4
23	Breath of Inferior ramus of the zygomatic process of maxillary	0.9 ± 0.2	1.9 ± 0.3	1.5 ± 0.2
24	Breath of base zygomatic process of squamosal	1.5 ± 0.2	3.0 ± 0.4	2.2 ± 0.4
25	Breadth of zygomatic plate	2.5 ± 0.2	5.1 ± 0.6	3.7 ± 0.6
26	Zygomatic breath	11.0 ± 0.5	22.4 ± 2.3	18.2 ± 1.0
27	Length of palatal foramen	4.1 ± 0.6	7.8 ± 0.8	6.1 ± 0.9
28	Smallest palatal breadth	2.1 ± 0.3	4.7 ± 0.6	3.6 ± 0.4
29	Upper cheek-teeth alveoli	3.4 ± 0.4	7.4 ± 0.4	6.7 ± 0.4
30	Breadth of upper dental arch	4.4 ± 0.2	9.4 ± 0.7	7.6 ± 0.4
31	Breadth of molar tooth 1	1.1 ± 0.1	2.8 ± 1.0	2.0 ± 0.2
32	Tympanic bulla length	2.4 ± 0.3	8.1 ± 0.6	7.1 ± 0.5
33	Tympanic bulla width	3.2 ± 0.1	6.0 ± 1.0	5.2 ± 0.6
34	Foramen magnum width	3.6 ± 0.3	6.9 ± 0.4	5.9 ± 0.3

* The parameters were measured in millimeters, *n*: Total observation, SD: Standard deviation of mean.

**Table 4:** Mandibular morphometric linear measurements (mean ± SD) of the commensal rodents (mean ± SD) rodents of Qatar.

Sl. No.	Parameters *	*Mus musculus* (*n* = 4)	*Rattus norvegicus* (*n* = 86)	*Rattus rattus* (*n* = 18)
1	Length of lower incisors	3.8 ± 0.5	9.5 ± 2.0	6.9 ± 1.2
2	Lower incisors to coronoid process	10.8 ± 0.1	25.4 ± 2.6	19.2 ± 2.2
3	Lower incisors to condylar process	13.4 ± 0.2	30.2 ± 2.9	23.4 ± 2.2
4	Lower incisors to angular process	13.5 ± 0.3	30.5 ± 3.1	23.7 ± 2.3
5	Greatest jaw height	6.6 ± 0.2	14.3 ± 1.6	11.1 ± 1.0
6	Ramus to molar tooth 1	3.8 ± 0.1	8.8 ± 1.0	6.6 ± 0.7
7	Lower molar tooth 1- molar tooth 3	3.3 ± 0.3	7.3 ± 0.3	6.3 ± 0.5
8	Lower incisors to molar tooth 1	5.0 ± 0.3	11.4 ± 1.4	8.6 ± 1.0

* The parameters were measured in millimeters; *n*: Total observation, SD: Standard deviation of mean.

**Table 5:** Sexual dimorphism of external body measurements (Mean ± SD) of *Rattus norvegicus* and *Rattus rattus*.

Sl. No.	Parameters *	*Rattus norvegicus*			*Rattus rattus*		
		Female (*n* = 62)	Male (*n* = 58)	*p*	Female (*n* = 10)	Male (*n* = 14)	*p*
1	Body weight	260.6 ± 76.1	268.2 ± 98.8	0.64	128.5 ± 65.7	131.0 ± 77.6	0.93
2	General length	396.5 ± 37.8	400.5 ± 52.0	0.63	342.5 ± 72.8	311.4 ± 85.0	0.36
3	Tail length	190.2 ± 18.8	192.7 ± 26.8	0.55	192.0 ± 42.0	173.6 ± 36.3	0.26
4	Body length	206.4 ± 20.1	207.8 ± 25.9	0.73	150.3 ± 33.1	137.9 ± 51.6	0.50
5	Right ear length	18.7 ± 1.7	19.0 ± 1.7	0.39	19.6 ± 1.8	17.9 ± 2.0	0.04
6	Right hind leg length	38.7 ± 2.9	39.8 ± 4.2	0.09	32.0 ± 2.0	32.6 ± 4.9	0.70

* The body weight was measured in grams and the rest of the parameters were measured in millimeters; *n*: Total observation, SD: Standard deviation of mean, and *p*: Probability at 95% confidence level.

**Table 6:** Sexual dimorphism of cranial morphometric measurements (mean ± SD) of *Rattus norvegicus* and *Rattus rattus*.

Sl. No.	Parameters *	*Rattus norvegicus*			*Rattus rattus*		
		Female (*n* = 38)	Male (*n* = 48)	*p*	Female (*n* = 9)	Male (*n* = 9)	*p*
1	General cranial length	46.3 ± 3.7	47.1 ± 4.3	0.38	38.2 ± 2.8	36.2 ± 2.2	0.11
2	Condylobasal length	44.8 ± 3.6	45.5 ± 4.4	0.48	35.3 ± 3.5	35.6 ± 2.3	0.83
3	Henselion-basion length	39.3 ± 3.5	38.9 ± 3.8	0.65	29.5 ± 3.8	29.2 ± 1.8	0.82
4	Henselion-palpation length	22.6 ± 1.9	22.2 ± 2.6	0.51	17.3 ± 1.4	16.4 ± 2.0	0.25
5	Length of upper incisor	7.5 ± 1.6	7.5 ± 1.6	0.95	5.8 ± 1.1	5.4 ± 0.9	0.38
6	Width of upper incisors, basal	4.8 ± 0.6	4.8 ± 0.6	0.77	3.6 ± 0.3	3.4 ± 0.7	0.40
7	Width of upper incisors, apex	3.2 ± 0.5	3.3 ± 0.4	0.31	2.4 ± 0.2	2.1 ± 0.4	0.08
8	Upper incisor to alveolus molar tooth 1	14.4 ± 1.8	14.4 ± 2.0	0.99	10.8 ± 1.3	9.5 ± 1.4	0.06
9	Length of diastema	13.3 ± 1.5	13.4 ± 1.5	0.77	10.2 ± 1.1	9.1 ± 1.2	0.06
10	Nasal bone length	17.2 ± 1.8	17.2 ± 2.0	0.89	13.0 ± 1.3	12.2 ± 1.4	0.24
11	Breath of nasal bones	5.1 ± 0.5	5.2 ± 0.6	0.30	4.0 ± 0.1	3.8 ± 0.4	0.38
12	Frontal bone length	14.6 ± 1.1	14.7 ± 1.5	0.72	12.8 ± 1.5	11.7 ± 1.1	0.13
13	Frontal bone width	10.8 ± 0.6	10.9 ± 0.6	0.55	10.4 ± 1.2	10.2 ± 0.9	0.63
14	Parietal bone length	13.0 ± 0.9	13.1 ± 1.2	0.75	11.6 ± 0.6	10.9 ± 1.2	0.16
15	Breath of brain cage	16.1 ± 2.0	16.6 ± 2.3	0.33	16.1 ± 0.6	16.3 ± 0.7	0.48
16	Interparietal bone length	6.6 ± 0.9	6.4 ± 0.6	0.24	5.6 ± 0.5	5.4 ± 0.7	0.51
17	Interparietal bone width	11.4 ± 1.0	11.6 ± 1.0	0.20	10.8 ± 0.8	10.6 ± 0.9	0.79
18	Occipital bone length	6.0 ± 0.7	6.0 ± 0.9	0.75	4.5 ± 0.5	4.5 ± 0.2	0.86
19	General cranial height	16.6 ± 1.6	16.6 ± 1.5	0.97	14.1 ± 0.7	13.2 ± 0.8	0.03
20	Rostrum height	13.8 ± 1.1	13.7 ± 1.4	0.72	11.2 ± 0.7	10.4 ± 1.0	0.07
21	Rostrum breathe	9.1 ± 0.9	9.0 ± 1.1	0.74	6.7 ± 0.6	6.4 ± 0.8	0.41
22	Smallest interorbital breadth	6.7 ± 0.4	6.9 ± 0.6	0.32	6.0 ± 0.4	5.6 ± 0.3	0.04
23	Breath of inferior ramus of the zygomatic process of maxillary	1.8 ± 0.3	1.9 ± 0.3	0.11	1.6 ± 0.2	1.4 ± 0.1	0.09
24	Breath of base zygomatic process of squamosal	2.9 ± 0.4	3.0 ± 0.4	0.70	2.3 ± 0.4	2.1 ± 0.5	0.40
25	Breadth of zygomatic plate	5.2 ± 0.5	5.0 ± 0.6	0.34	4.0 ± 0.3	3.5 ± 0.8	0.08
26	Zygomatic breath	22.4 ± 2.0	22.3 ± 2.5	0.82	18.5 ± 0.8	17.9 ± 1.2	0.25
27	Length of palatal foramen	7.9 ± 0.7	7.8 ± 0.9	0.60	6.0 ± 1.0	6.1 ± 0.8	0.69
28	Smallest palatal breadth	4.8 ± 0.6	4.6 ± 0.5	0.29	3.8 ± 0.4	3.4 ± 0.4	0.06
29	Upper cheek-teeth alveoli	7.3 ± 0.4	7.4 ± 0.4	0.16	6.6 ± 0.4	6.7 ± 0.4	0.55
30	Breadth of upper dental arch	9.4 ± 0.7	9.4 ± 0.7	0.57	7.8 ± 0.3	7.5 ± 0.5	0.19
31	Breadth of molar tooth 1	2.8 ± 1.0	2.7 ± 1.0	0.84	2.0 ± 0.3	2.0 ± 0.2	0.65
32	Tympanic bulla length	8.1 ± 0.5	8.1 ± 0.7	0.76	7.1 ± 0.6	7.1 ± 0.5	0.91
33	Tympanic bulla width	6.1 ± 1.1	6.0 ± 0.9	0.78	5.2 ± 0.7	5.2 ± 0.6	0.99
34	Foramen magnum width	6.8 ± 0.4	6.9 ± 0.4	0.08	6.0 ± 0.3	5.8 ± 0.2	0.39

* The parameters were measured in millimeters; *n*: Total observation, SD: Standard deviation of mean, and *p*: Probability at 95% confidence level.

**Table 7:** Sexual dimorphism of mandibular morphometric measurements (mean ± SD) of *Rattus norvegicus* and *Rattus rattus*.

Sl. No.	Parameters *	*Rattus norvegicus*			*Rattus rattus*		
		Female (*n* = 38)	Male (*n* = 48)	*p*	Female (*n* = 9)	Male (*n* = 9)	*p*
1	Length of lower incisors	9.4 ± 1.9	9.6 ± 2.2	0.73	7.6 ± 1.1	6.3 ± 1.1	0.02
2	Lower incisors to coronoid process	25.1 ± 2.1	25.6 ± 2.9	0.34	20.6 ± 1.3	17.7 ± 1.9	0.01
3	Lower incisors to condylar process	30.4 ± 3.0	30.0 ± 2.9	0.57	24.8 ± 1.3	22.1 ± 2.1	0.01
4	Lower incisors to angular process	30.6 ± 3.0	30.4 ± 3.2	0.69	25.0 ± 1.4	22.3 ± 2.3	0.01
5	Greatest jaw height	14.4 ± 1.6	14.3 ± 1.6	0.71	11.6 ± 1.0	10.7 ± 0.8	0.06
6	Ramus to molar tooth 1	8.8 ± 1.1	8.7 ± 0.9	0.70	7.1 ± 0.5	6.2 ± 0.6	0.01
7	Lower molar tooth 1- molar tooth 3	7.3 ± 0.3	7.3 ± 0.3	0.84	6.3 ± 0.4	6.4 ± 0.7	0.80
8	Lower incisors to molar tooth 1	11.3 ± 1.2	11.4 ± 1.5	0.63	9.3 ± 0.8	8.0 ± 0.8	0.01

* The parameters were measured in millimeters; *n*: Total observation, SD: Standard deviation of mean, and *p*: Probability at 95% confidence level.

**Table 8:** Pregnancy-related external body morphometric dimorphism (mean ± SD) in *Rattus norvegicus* and *Rattus rattus*.

Sl. No.	Parameters *	*Rattus norvegicus*			*Rattus rattus*		
		Pregnant (*n* = 16)	Non-Pregnant (*n* = 45)	*p*	Pregnant (*n* = 8)	Non-Pregnant (*n* = 2)	*p*
1	Body weight	275.3 ± 88.0	260.0 ± 65.6	0.47	111.5 ± 28.9	196.5 ± 146.4	0.10
2	General length	400.6 ± 31.1	398.3 ± 33.9	0.81	348.8 ± 68.1	317.5 ± 116.7	0.62
3	Tail length	190.6 ± 15.5	191.6 ± 17.1	0.85	196.9 ± 42.8	172.5 ± 46.0	0.50
4	Body length	210.0 ± 17.6	206.8 ± 17.7	0.53	151.9 ± 26.2	145.0 ± 70.1	0.81
5	Right ear length	18.9 ± 1.9	18.7 ± 1.7	0.78	19.8 ± 2.0	19.0 ± 1.4	0.63
6	Right hind leg length	38.3 ± 3.6	39.0 ± 2.3	0.37	31.4 ± 1.1	34.5 ± 3.5	0.04

* Body weight was measured in grams and rest of the parameters were measured in millimeters; *n*: Total observation, SD: Standard deviation of mean, and *p*: Probability at 95% confidence level.

**Table 9:** Pregnancy-related cranial morphometric dimorphism (mean ± SD) in *Rattus norvegicus* and *Rattus rattus*.

Sl. No.	Parameters *	*Rattus norvegicus*			*Rattus rattus*		
		Pregnant (*n* = 11)	Non-Pregnant (*n* = 27)	*p*	Pregnant (*n* = 7)	Non-Pregnant (*n* = 2)	*p*
1	General cranial length	46.0 ± 3.8	46.5 ± 3.8	0.74	38.7 ± 3.1	36.7 ± 0.3	0.41
2	Condylobasal length	44.6 ± 3.8	45.0 ± 3.6	0.77	36.0 ± 3.8	33.0 ± 1.4	0.34
3	Henselion-basion length	39.4 ± 3.4	39.2 ± 3.6	0.88	30.2 ± 3.8	27.2 ± 4.2	0.36
4	Henselion-palpation length	22.7 ± 2.1	22.5 ± 1.9	0.76	17.4 ± 1.6	17.0 ± 0.8	0.73
5	Length of upper incisor	7.8 ± 1.0	7.4 ± 1.9	0.53	5.8 ± 1.3	5.8 ± 0.1	0.99
6	Width of upper incisors, basal	4.7 ± 0.7	4.8 ± 0.6	0.75	3.6 ± 0.2	3.6 ± 0.5	0.66
7	Width of upper incisors, apex	3.1 ± 0.5	3.2 ± 0.5	0.47	2.4 ± 0.2	2.2 ± 0.1	0.19
8	Upper incisor to alveolus molar tooth 1	14.7 ± 1.1	14.2 ± 2.0	0.43	11.0 ± 1.2	9.8 ± 1.2	0.26
9	Length of diastema	13.3 ± 1.6	13.3 ± 1.4	0.94	10.4 ± 1.1	9.4 ± 0.8	0.29
10	Nasal bone length	17.2 ± 2.0	17.2 ± 1.8	0.97	13.3 ± 1.4	12.2 ± 0.3	0.32
11	Breath of nasal bones	5.1 ± 0.5	5.1 ± 0.6	0.90	4.0 ± 0.2	4.1 ± 0.1	0.32
12	Frontal bone length	14.3 ± 1.0	14.7 ± 1.2	0.27	12.9 ± 1.7	12.2 ± 0.7	0.61
13	Frontal bone width	10.9 ± 0.6	10.8 ± 0.6	0.87	10.7 ± 1.3	9.4 ± 0.1	0.19
14	Parietal bone length	12.9 ± 0.3	13.1 ± 1.1	0.61	11.6 ± 0.7	11.5 ± 0.3	0.83
15	Breath of brain cage	16.5 ± 0.9	16.0 ± 2.3	0.49	16.1 ± 0.7	16.0 ± 0.4	0.81
16	Interparietal bone length	6.6 ± 1.0	6.6 ± 0.8	0.84	5.6 ± 0.5	5.8 ± 0.6	0.59
17	Interparietal bone width	11.0 ± 0.8	11.5 ± 1.0	0.19	10.8 ± 0.8	10.5 ± 1.3	0.59
18	Occipital bone length	5.8 ± 0.8	6.1 ± 0.6	0.20	4.4 ± 0.5	4.9 ± 0.3	0.21
19	General cranial height	16.3 ± 1.6	16.7 ± 1.7	0.49	14.0 ± 0.7	14.3 ± 0.9	0.67
20	Rostrum height	13.6 ± 1.3	13.9 ± 1.1	0.41	11.2 ± 0.8	11.0 ± 0.2	0.77
21	Rostrum breathe	9.0 ± 0.9	9.1 ± 0.9	0.68	6.8 ± 0.7	6.5 ± 0.2	0.61
22	Smallest interorbital breadth	6.6 ± 0.5	6.8 ± 0.4	0.21	6.1 ± 0.5	5.8 ± 0.1	0.44
23	Breath of inferior ramus of the zygomatic process of maxillary	1.8 ± 0.3	1.9 ± 0.3	0.26	1.6 ± 0.3	1.5 ± 0.1	0.63
24	Breath of base zygomatic process of squamosal	3.0 ± 0.5	2.9 ± 0.4	0.63	2.4 ± 0.4	2.0 ± 0.2	0.21
25	Breadth of zygomatic plate	5.2 ± 0.4	5.2 ± 0.5	0.89	4.0 ± 0.4	4.1 ± 0.2	0.63
26	Zygomatic breath	22.5 ± 1.7	22.4 ± 2.2	0.91	18.6 ± 0.8	17.9 ± 0.6	0.34
27	Length of palatal foramen	7.9 ± 0.7	7.9 ± 0.6	0.82	5.9 ± 1.2	6.1 ± 0.1	0.88
28	Smallest palatal breadth	4.7 ± 0.6	4.8 ± 0.5	0.54	3.9 ± 0.4	3.3 ± 0.2	0.06
29	Upper cheek-teeth alveoli	7.3 ± 0.5	7.4 ± 0.3	0.41	6.5 ± 0.4	6.9 ± 0.4	0.21
30	Breadth of upper dental arch	9.3 ± 0.8	9.5 ± 0.7	0.53	7.8 ± 0.3	7.8 ± 0.4	0.78
31	Breadth of molar tooth 1	2.8 ± 1.1	2.8 ± 1.0	0.98	2.0 ± 0.3	2.2 ± 0.2	0.26
32	Tympanic bulla length	8.1 ± 0.4	8.1 ± 0.5	0.99	7.2 ± 0.7	6.6 ± 0.1	0.25
33	Tympanic bulla width	6.1 ± 1.3	6.0 ± 1.0	0.77	5.4 ± 0.6	4.4 ± 0.2	0.05
34	Foramen magnum width	6.5 ± 0.4	6.9 ± 0.4	0.05	38.7 ± 3.1	6.1 ± 0.5	0.47

* The parameters were measured in millimeter; *n*: Total observation, SD: Standard deviation of mean, and *p*: Probability at 95% confidence level.

**Table 10:** Pregnancy-related mandibular morphometric dimorphism (mean ± SD) in *Rattus norvegicus* and *Rattus rattus*.

Sl. No.	Parameters *	*Rattus norvegicus*			*Rattus rattus*		
		Pregnant (*n* = 11)	Non-Pregnant (*n* = 27)	*p*	Pregnant (*n* = 7)	Non-Pregnant (*n* = 2)	*p*
1	Length of lower incisors	9.5 ± 1.1	9.4 ± 2.2	0.90	7.7 ± 1.2	7.2 ± 0.4	0.64
2	Lower incisors to coronoid process	24.9 ± 2.2	25.1 ± 2.1	0.75	20.9 ± 1.3	19.6 ± 0.5	0.19
3	Lower incisors to condylar process	30.1 ± 2.6	30.5 ± 3.1	0.70	25.1 ± 1.2	23.4 ± 0.3	0.10
4	Lower incisors to angular process	30.3 ± 2.8	30.8 ± 3.1	0.63	25.5 ± 1.2	23.4 ± 0.1	0.05
5	Greatest jaw height	14.0 ± 1.5	14.6 ± 1.6	0.35	11.8 ± 1.0	10.8 ± 0.8	0.23
6	Ramus to molar tooth M1	8.5 ± 1.2	9.0 ± 1.0	0.28	7.2 ± 0.5	6.7 ± 0.3	0.24
7	Lower molar tooth M1- molar tooth 3	7.2 ± 0.3	7.4 ± 0.3	0.13	6.2 ± 0.4	6.6 ± 0.1	0.19
8	Lower incisors to molar tooth 1	11.1 ± 1.1	11.4 ± 1.3	0.57	9.5 ± 0.7	8.4 ± 0.8	0.12

* The parameters were measured in millimeters; *n*: Total observation, SD: Standard deviation of mean, and *p*: Probability at 95% confidence level.

## Data Availability

All the data are available with the first author, can be delivered if required.
